# IL-18-Mediated SLC7A5 Overexpression Enhances Osteogenic Differentiation of Human Bone Marrow Mesenchymal Stem Cells *via* the c-MYC Pathway

**DOI:** 10.3389/fcell.2021.748831

**Published:** 2021-12-17

**Authors:** Feifei Ni, Tao Zhang, Wanan Xiao, Hong Dong, Jian Gao, YaFeng Liu, Jianjun Li

**Affiliations:** ^1^ Department of Orthopaedics, Shengjing Hospital of China Medical University, Shenyang, China; ^2^ Liaoning Qifu Stem Cell Biotechnology Co, Ltd, Shenyang, China

**Keywords:** IL-18, human bone marrow mesenchymal stem cells, c-Myc, osteogenic differentiation, SLC7A5

## Abstract

**Objective:** To investigate the role of IL-18 in the regulation of osteogenic differentiation in human bone marrow mesenchymal stem cells (hBMSCs).

**Methods:** To assess whether IL-18 affects the osteogenic differentiation of hBMSCs through the c-MYC/SLC7A5 axis, IL-18 dose-response and time-course experiments were performed to evaluate its impact on osteogenic differentiation. To confirm osteogenic differentiation, alizarin red staining calcium measurement were performed. RT-qPCR and western blotting were used to determine the expression levels of bone-specific markers ALP, RUNX2, and BMP2, as well as those of SLC7A5 and c-MYC. Furthermore, SLC7A5 and c-MYC expression was evaluated *via* immunofluorescence. To elucidate the roles of SLC7A5 and c-MYC in osteoblast differentiation, cells were transfected with SLC7A5 or c-MYC siRNAs, or treated with the SLC7A5-specific inhibitor JPH203 and c-MYC-specific inhibitor 10058-F4, and the expression of SLC7A5, c-MYC, and bone-specific markers ALP, RUNX2, and BMP2 was assessed.

**Results:** Our results demonstrated that IL-18 increased calcium deposition in hBMSCs, and upregulated the expression of SLC7A5, c-MYC, ALP, RUNX2, and BMP2. Silencing of SLC7A5 or c-MYC using siRNA reduced the expression of ALP, RUNX2, and BMP2, while IL-18 treatment partially reversed the inhibitory effect of siRNA. Similar results were obtained by treating hBMSCs with SLC7A5 and c-MYC specific inhibitors, leading to significant reduction of the osteogenesis effect of IL-18 on hBMSCs.

**Conclusion:** In conclusion, our results indicate that IL-18 promotes the osteogenic differentiation of hBMSCs *via* the SLC7A5/c-MYC pathway and, therefore, may play an important role in fracture healing. These findings will provide new treatment strategies for delayed fracture healing after splenectomy.

## Introduction

In a preliminary clinical study, we previously demonstrated that fracture healing was significantly delayed in patients undergoing splenectomy, while inflammatory factors were significantly reduced during the acute phase (Xiao et al., 2017; Xiao et al., 2018). These results indicated that during certain stages of bone healing, the inflammatory immune response at the fracture site is indispensable (Claes et al., 2012). However, the specific role of pro-inflammatory factors during fracture healing is still not clear.

Early inflammatory response is a key aspect of fracture repair. The administration of anti-inflammatory drugs, such as steroids, at the early stage of fracture healing is not advantageous based on reported outcomes (Chan et al., 2015). In response to a fracture, the inflammatory microenvironment formed around the edges of the fracture recruits bone marrow mesenchymal stem cells (BMSCs) to the site to participate in fracture repair (Reich et al., 2020). In this microenvironment, BMSCs express numerous cytokines and induce the release of a series of cytokines, leading to the activation of autocrine and paracrine pathways, BMSC homing, and osteoblast differentiation, which is beneficial to fracture repair (Zhao et al., 2020). As inflammation subsides, mesenchymal stem cells (MSCs) and other progenitor cells proliferate and form granulation tissue, which is eventually converted to a cartilaginous callus to stabilize the fracture site.

Interleukin 18 (IL-18), a pro-inflammatory cytokine, has been shown to enhance the activity of natural killer (NK) cells, T cells, B cells, and macrophages in the spleen to produce interferon-γ (INF-γ), and plays an important role in inflammation and immune responses (Apalset et al., 2014). Monocytes, macrophages, osteoblasts, BMSCs, and other cells can also express IL-18 (Vecchié et al., 2021). Furthermore, it has been reported that IL-18 can inhibit collagen synthesis and regulate osteoblast differentiation and function. IL-18 expression has been detected in several organs and tissues, such as liver, kidney, spleen, pancreas, lungs, and skeletal muscles. IL-18 plays an important role in fracture repair by coordinating the homing of BMSCs, as well as the differentiation of osteoblasts and osteoclasts; however, the exact mechanism is still unclear (Gibon et al., 2016; Lima Leite et al., 2020).

Amino acids play an important role in the maintenance of cellular homeostasis and various physiological and biochemical processes in cells. Solute carrier family 7, member 5 (SLC7A5) is a Na^+^ and pH-independent large neutral amino acid transporter involved in the uptake of essential amino acids, such as leucine, phenylalanine, and valine. The gene is located on human chromosome 16, contains 39,477 nucleotides and 10 exons, and encodes a 507 amino acid 12-pass transmembrane protein with a molecular mass of 55 kDa (Singh and Ecker, 2018). SLC7A5 is highly expressed in the spleen, bone marrow, and placenta, as well as by monocytes and macrophages. SLC7A5 can act as a cellular nutrient signal receptor by regulating the transport of amino acids and providing key components for numerous cellular processes, such as cell proliferation and differentiation (Kandasamy et al., 2018). It has been recently reported that IL-18 can upregulate the expression of SLC7A5 in NK cells, and that amino acid transport mediated by SLC7A5 has an impact on the expression levels of c-MYC. When SLC7A5 is inhibited, c-MYC protein levels drop rapidly, leading to a significant reduction in NK cell metabolism (Liu et al., 2017; Almutairi et al., 2019). Furthermore, research shows that c-MYC expression in T cells is affected by the intracellular amino acid levels (Loftus et al., 2018). Studies have shown that SLC7A5 and c-MYC are highly expressed in stem cells and play roles in most tissues and cells where terminal differentiation occurs (Melnik et al., 2019; Poncet et al., 2020). However, the roles of SLC7A5 and c-MYC in the osteogenic differentiation of human BMSCs (hBMSCs) has not been reported.

Based on these findings, we hypothesized that IL-18 regulates the osteogenic differentiation of hBMSCs *via* the SLC7A5/c-MYC axis. In this study, we investigated the potential roles of IL-18, SLC7A5, and c-MYC during osteogenic differentiation.

## Materials and Methods

### Cell Culture

hBMSCs were provided by the Liaoning Qifu Stem Cell Biotechnology Co, Ltd, China. Cells were cultured in DMEM/F12 (Heyclone, United States) medium supplemented with 15% FBS (Excell Bio, China) and 1% penicillin/streptomycin (Sigma, United States), and passage 3 cells were used for all experiments. To induce osteogenic differentiation, hBMSCs were plated at a density of 10×10^4^ cells in 6-well plates and cultured in the classical osteogenic differentiation medium (DMEM/F12 containing 15% FBS and 1% penicillin/streptomycin and supplemented with 100 nM dexamethasone (Sigma, United States), 10 mM β-glycerophosphate (Sigma, United States), and 50 μM ascorbic acid (Sigma, United States)). Cells were cultured in a 37°C, 5% CO_2_ incubator, and the medium was changed every 2 days. The cells were seeded in a six-well plate for osteogenic differentiation at 150,000 per well. To evaluate the effect of recombinant human IL-18 (R&D, United States) on osteogenic differentiation, the cells were treated with different concentrations of IL-18 (0 ng/ml as control, 1, 10, 50, 100 ng/ml) for 12 h. In another set of experiments, cells were treated with 100 ng/ml IL-18 and incubated for 0 (control), 3, 6, 12, and 24 h. Based on the results of these dose-response and time-course experiments, we selected 100 ng/ml as the IL-18 concentration for all subsequent experiments. To investigate the involvement of the SLC7A5/c-MYC axis, hBMSCs were pretreated with SLC7A5 specific inhibitor JPH203 (5 μM) (MCE, United States) and c-MYC specific inhibitor 10058-F4 (5 μM) (APExBIO, United States) for 48 or 72 h, and then cultured with IL-18 (100 ng/ml) for 12 h. JPH203, and 10058-F4 were dissolved in DMSO (final concentration of DMSO<0.01%)

### Alizarin Red Staining and Calcium Measurement

To confirm the formation of calcium deposits, hBMSCs were cultured in osteogenic medium for 7 days. Next, the cells were fixed with 4% paraformaldehyde for 20 min, washed 3 times with PBS, stained with Alizarin Red (Sigma, United States) for 20 min at room temperature, washed 3 times with PBS, and then observed under the microscope. Then use the Calcium Colorimetric Assay Kit (Beyotime, China) for calcium measurement. Follow the instructions. In short, wash and dilute the cells and extracellular matrix in a buffer solution, and add the active solution to each well. After 15 min of incubation in the dark at room temperature, the calcium concentration is measured at 575 nm wavelength.

### RNA Extraction and Reverse Transcription Quantitative Real-Time Polymerase Chain Reaction

Total RNA was extracted from BMSCs cultured in osteogenic medium using RNAiso Plus reagent (9108, Takara, Japan). Samples with optical density (OD) 260/280 nm between 1.8 and 2.0 were used for cDNA synthesis. Reverse transcription was conducted using a PrimeScript RT Reagent Kit (RR037A, Takara). Quantitative PCR was performed on an ABI 7500fast System (Life Technologies, United States) using TB Green™ Premix Ex Taq ™II (RR820A, Takara) according to the manufacturer’s instructions. conditions of real-time PCR were as follows: denaturation at 95°C for 30 s, 40 cycles at 95°C for 3 s and 60°C for 30 s, The dissociation stage was added to the end of the amplification procedure. The following primers (Takara) were used:

*SLC7A5* forward: 5′-GCA​TCG​GCT​TCA​CCA​TCA​TC-3′,

reverse: 5′-ACC​ACC​TGC​ATG​AGC​TTC​TGA​C-3′;

*c-MYC* forward: 5′-GGA​GGC​TAT​TCT​GCC​CAT​TTG-3′,

reverse: 5′-CGA​GGT​CAT​AGT​TCC​TGT​TGG​TG-3′;

*ALP* forward: 5′-CAA​ATG​CCT​GGA​TCC​TGT​TGA​C-3′,

reverse: 5′-TGC​ACT​GGC​CAT​CCA​TCT​C-3′;

*RUNX2* forward: 5′-CAC​TGG​CGC​TGC​AAC​AAG​A-3′,

reverse: 5′-CAT​TCC​GGA​GCT​CAG​CAG​AAT​AA-3';

*BMP2* forward: 5′-AAC​ACT​GTG​CGC​AGC​TTC​C-3′

reverse: 5′-CCT​AAA​GCA​TCT​TGC​ATC​TGT​TCT​C-3';

*β-actin* forward: 5′-TGG​CAC​CCA​GCA​CAA​TGA​A-3′

reverse: 5′-CTA​AGT​CAT​AGT​CCG​CCT​AGA​AGC​A-3'.

Gene expression analyses were calculated using the 2^−ΔΔCt^ method.

### siRNA and Transfection

Small interfering RNAs (siRNAs) targeting SLC7A5 (5′-GCG​UCA​UGU​CCU​GGA​UCA​UTT​AUG​AUC​CAG​GAC​AUG​ACG​CTT-3′), c-MYC (5′-CAC​CUA​UGA​ACU​UGU​UUC​ATT​UGA​AAC​AAG​UUC​AUA​GGU​GTT-3′), and the negative control (NC, 5′-UUC​UCC​GAA​CGU​GUC​ACG​UTT-3′) were purchased from Genepharma (Suzhou, China). Cells were transfected with 75 nmol/L of either SLC7A5 siRNA, c-MYC siRNA, or NC siRNA using Lipofectamine™ 3000 Transfection Reagent (Thermo Scientific, United States) according to the manufacturer’s guidelines. The cells were collected for RNA or protein isolation 24–72 h post-transfection to evaluate the effects of the treatment on osteogenic differentiation.

### Western Blotting

Cells were cultured as described above, washed with PBS, and scraped off the culture plates, and the total cell protein was extracted with radioimmunoprecipitation assay buffer containing protease and phosphatase inhibitors according to the instructions. The protein concentration was determined using the bicinchoninic acid protein assay (Biyotime, China). Equal amounts of protein/sample (20 μg) were separated using sodium dodecyl-sulfate polyacrylamide gel electrophoresis (SDS-PAGE) and then transferred to a polyvinylidene difluoride membrane (Millipore, United States). The membranes were blocked in 5% skim milk in tris-buffered saline with Tween 20 (TBST) and then incubated with primary antibodies at 4°C overnight. The following primary antibodies were used: GAPDH (60004-1-Ig, Proteintech, United States), SLC7A5 (13752-1-AP, Proteintech), c-MYC (10828-1-AP, Proteintech), ALP (11187-1-AP, Proteintech), BMP2 (18933-1-AP, Proteintech), RUNX2 (AF5186, Affinity, United States). All primary antibodies were diluted according to the manufacturers’ instructions. Next, the membranes were washed 3 × 15 min with TBST and then incubated with a secondary antibody goat anti-rabbit IgG (HRP-6004, Proteintech) at room temperature for 2 h. Finally, the target bands were visualized using the enhanced chemiluminescence reagent (Biosharp, China) and a Plus Western Blotting Detection System (GE680, United States).

### Immunofluorescence Staining

In a 24-well plate, the slides that have climbed up cells were washed 3 × 3 min in PBS, fixed with 4% paraformaldehyde for 15 min, permeabilized with 0.5% Triton X-100 in PBS preparation at room temperature 20 min, and then blocked with normal goat serum for 30 min at room temperature. The blocking solution was removed using the absorbent paper and a sufficient amount of diluted anti-SLC7A5 or anti-c-MYC primary antibody was added to each slide. Slides were incubated overnight at 4°C in a humidified container. Next, the cells were incubated with CoraLite488-conjugated secondary antibody (1:100, SA00013-2, Proteintech) or CoraLite594-conjugated secondary antibody (1:100, SA00013-4, Proteintech) for 2 h at room temperature. 4′,6-Diamidino-2-phenylindole was added dropwise, and the cells were incubated for 5 min in the dark to stain the nuclei. The slides were mounted using an anti-quenching mounting solution, and the cells were observed under an inverted phase contrast microscope and image acquisition system for observing and acquiring images (Eclipse NI, Nikon).

### Statistical Analysis

Statistical analyses were conducted using the SPSS software version 16.0 (SPSS Inc, United States). All quantitative data are expressed as the mean ± standard deviation (SD) of three independent experiments. Differences between groups were analyzed using One-way ANOVA with a subsequent Bonferroni post-hoc test. *p* < 0.05 was considered statistically significant.

## Results

### IL-18 Enhances Calcium Deposition

**FIGURE 1:**
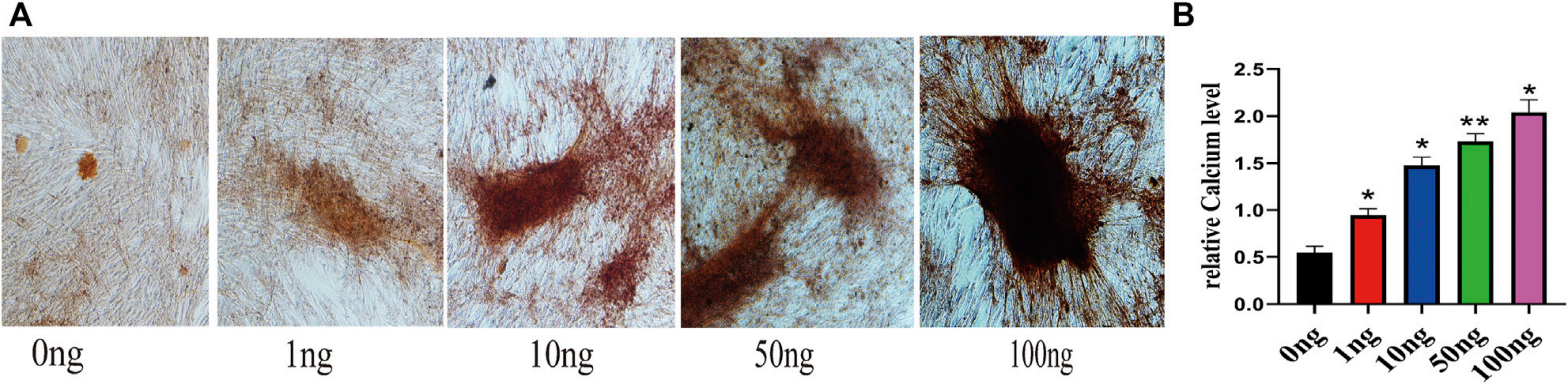
IL-18 promotes the osteogenic differentiation of hBMSCs. hBMSCs were treated with different concentrations of IL-18 for 7 days and calcium deposition was evaluated using Alizarin Red staining **(A)** and quantitative calcium assay is used to measure calcium concentration **(B)**. Data are expressed as the mean ± standard deviation (SD). Concentration groups vs. the control (0 ng) group. Experiments were repeated three times. Scale bars: 200 μm for A, ∗*p* < 0.05, ∗∗*p* < 0.01.

To investigate the effect of IL-18 on osteogenesis in hBMSCs *in vitro*, Alizarin Red staining was performed. Mineralization is commonly used as a late marker of osteogenesis; therefore, the cells were evaluated on day 7 after osteogenic induction by Alizarin red staining. Alizarin red staining showed that the number of mineralized nodules also increased significantly at higher IL-18 concentrations, with the highest staining intensity being observed at 100 ng/ml IL-18 (Figure 1A). According to the quantitative analysis of calcium, Compared with 0ng group hBMSCs cultured in osteogenic induction medium showed that the amount of calcium deposition increased with the concentration (Figure 1B).

### IL-18 Promotes the Differentiation of hBMSCs Into Osteoblasts in a Time- and Dose-dependent Manner

**FIGURE 2:**
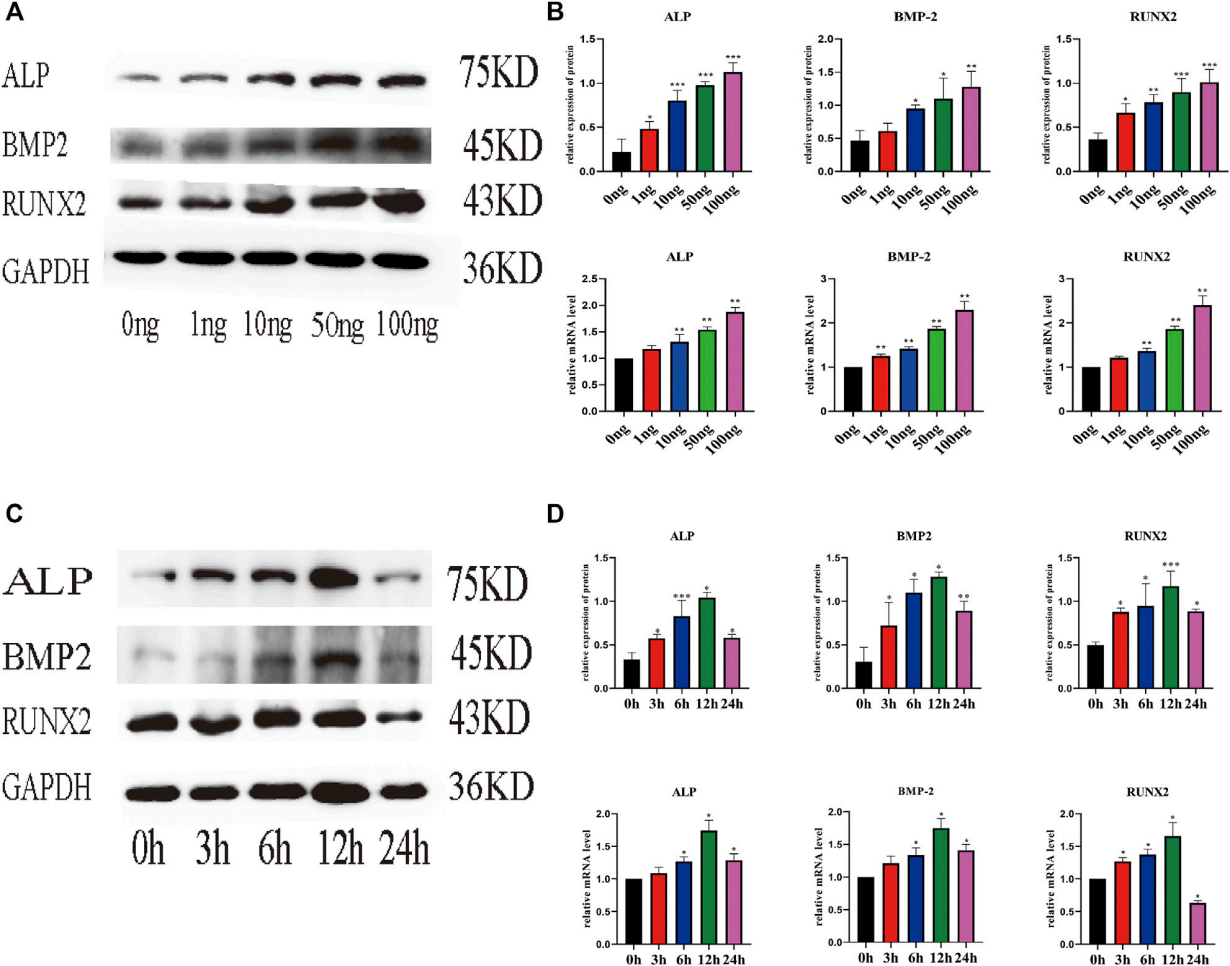
IL-18 induced the expression of osteoblast-specific markers in a dose- and time-dependent manner. hBMSCs were treated with different culture concentrations (0, 1, 10, 50, 100 ng) of IL-18, western blot and qPCR were used to evaluate the protein and mRNA **(A, B)** expression levels of ALP, BMP2, RUNX2, GAPDH. hBMSCs were treated with 100 ng/ml IL-18 for 0, 3, 6, 12 and 24 h, and Western Blotting and qPCR were used to evaluate the protein expression of osteoblast-specific markers ALP, BMP2, RUNX2, GAPDH and their mRNA expression **(C, D)**. GAPDH was used as an internal control. Data are expressed as the mean ± standard deviation (SD). Concentration groups vs. the control (0 ng) group; time-point groups vs. the control (0 h) group, *p < 0.05, **p < 0.01, ***p < 0.001.

Real-time PCR (qPCR) and WB were used to evaluate the effect of IL-18 on the expression of osteoblast-specific markers. IL-18 increased the mRNA expression of ALP, BMP2, and RUNX2 in a dose-dependent manner, with the highest expression levels being observed at the 100 ng/ml IL-18 dose. Similar results were observed in WB experiments (Figures 2A,B). Next, we performed time-course experiments (3–24 h) to assess the effect of IL-18 (100 ng/ml dose) on the expression of ALP, BMP2, and RUNX2 in hBMSCs. Our results demonstrated that BMP2 expression started to increase at the 6 h time-point, while ALP and RUNX2 expression started to increase at the 3 h time-point. The expression levels of all markers were the highest at 12 h and decreased by 24 h (Figures 2C,D).

### IL-18 Promotes the Increase of c-MYC Expression *via* SLC7A5 in a Time- and Dose-dependent Manner

**FIGURE 3:**
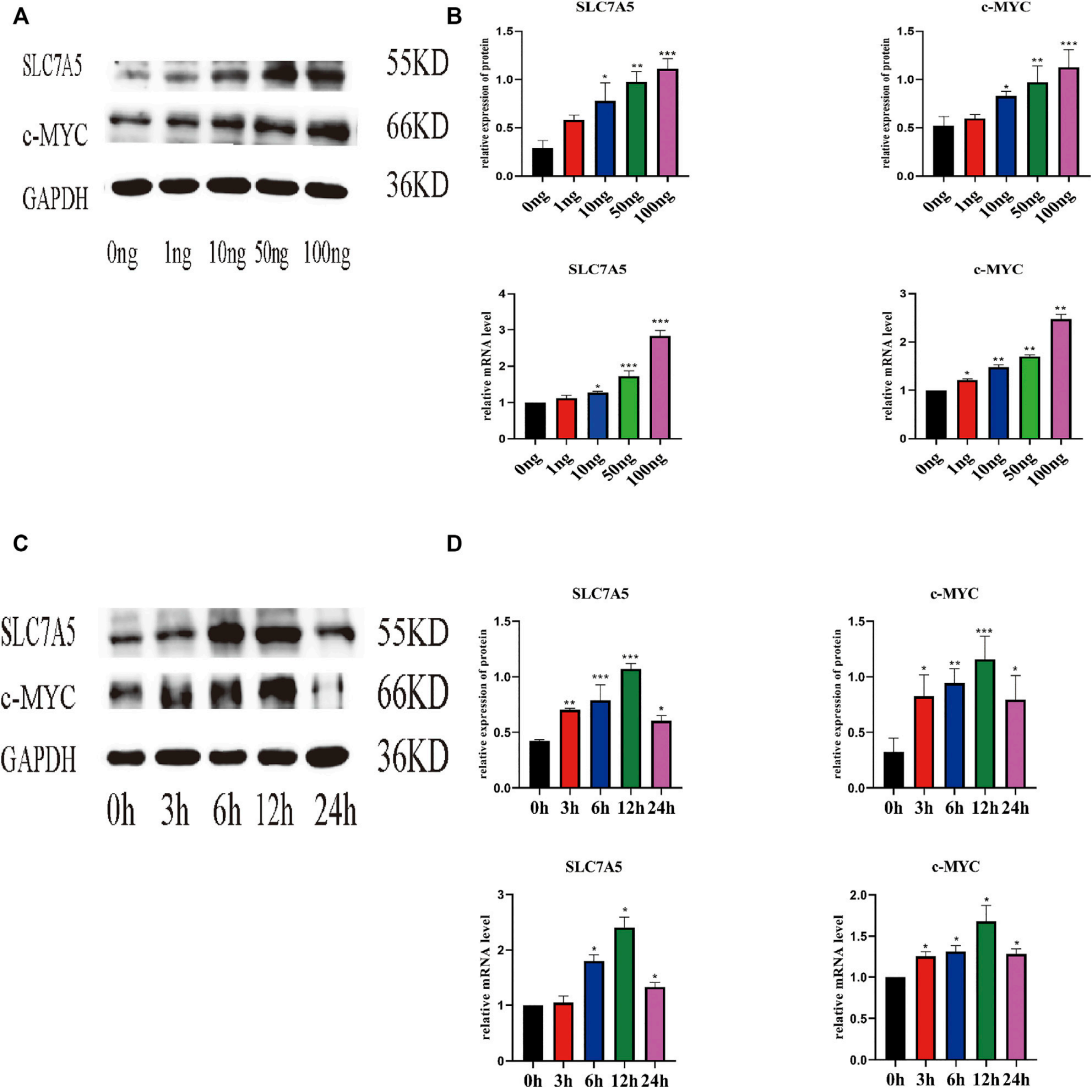
IL-18 induced the expression of SLC7A5 and –MYC in a dose- and time-dependent manner. hBMSCs were treated with different culture concentrations (0, 1, 10, 50, 100 ng) of IL-18, western blot and qPCR were used to evaluate the protein and mRNA **(A, B)** expression levels of SLC7A5, c-MYC, and GAPDH. hBMSCs were treated with 100 ng/ml IL-18 for 0, 3, 6, 12, and 24 h, and Western Blotting and qPCR were used to evaluate the protein expression of SLC7A5,c-MYC and GAPDH (as internal control) and their mRNA expression **(C, D)**. Data are expressed as the mean ± standard deviation (SD). Concentration groups vs. the control (0 ng) group; time-point groups vs. the control (0 h) group, Experiments were repeated three times. *p < 0.05, **p < 0.01, ***p < 0.001.

Next, we evaluated the effect of IL-18 on the expression of SLC7A5 and c-MYC using qPCR and western blot. qPCR results showed that IL-18 affected SLC7A5 and c-MYC expression in a dose-dependent manner; SLC7A5 expression started to increase at 10 ng/ml, while c-MYC expression was induced at 1 ng/ml, and the expression of both genes reached maximum levels at 100 ng/ml (Figures 3A,B). Time-course experiments demonstrated that SLC7A5 expression was initiated at the 6-h time point, while c-MYC expression was induced at the 3 h time point, and the expression of both genes reached their highest levels by 12 h. Western blot results confirmed these findings; the protein expression levels of SLC7A5 and c-MYC were the highest at 100 ng/ml dose, while time-course experiments showed that the protein expression levels of SLC7A5 and c-MYC were upregulated at 3 h, reached the maximum levels at 12 h, and decreased by 24 h (Figures 3C,D).

### Determination of Optimal Concentrations for SLC7A5 and c-MYC Inhibitors

**FIGURE 4:**
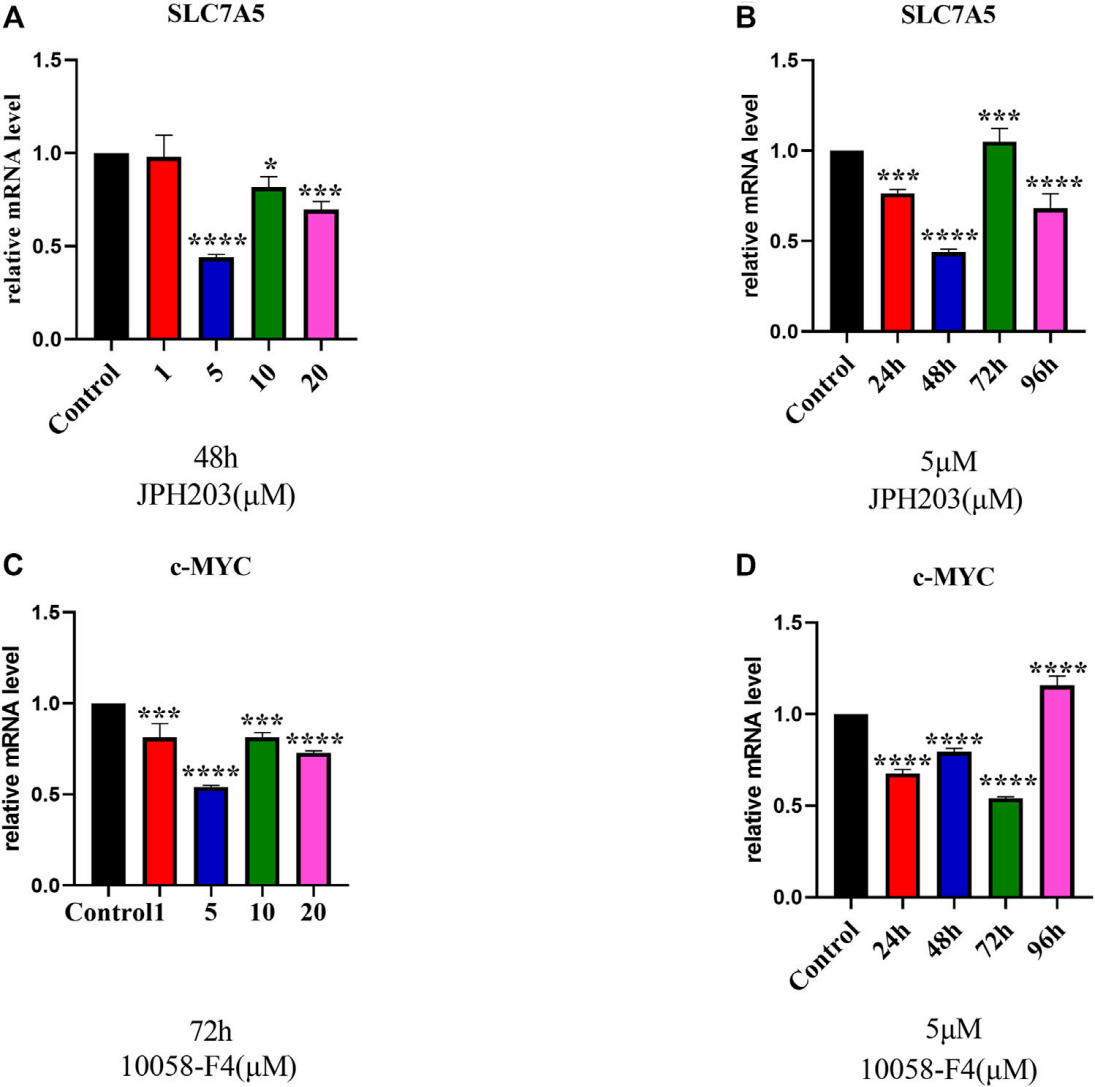
RT-qPCR evaluation of SLC7A5 **(A, B)** and c-MYC **(C, D)** gene expression. hBMSCs were cultured in the osteogenesis induction medium and then treated 10058-F4 and JPH203 with different concentrations (0, 1, 5, 10, 20 μM) and for different time periods (0, 24, 48, 72, 96 h). GAPDH was used as an internal control. Data are presented as the mean ± SEM, Concentration groups vs. the control group; time-point groups vs. the control group. Experiments were repeated three times. *p < 0.05, **p < 0.01, ***p < 0.001, ****p < 0.0001.

To evaluate the involvement of SLC7A5 and c-MYC in osteoblast differentiation, first, we determined the optimal concentration of JPH203 (a specific SLC7A5 inhibitor) and 10058-F4 (a specific c-MYC inhibitor) using real-time PCR. The cells were treated with different concentrations of JPH203 for 48 h and 10058-F4 (5 μM) for 72 h. SLC7A5 and c-MYC mRNA expression decreased significantly (Figures 4A,B).

### Osteogenic Differentiation Ability of hBMSCsis Reduced by SLC7A5 Inhibition and Enhanced by IL-18

**FIGURE 5:**
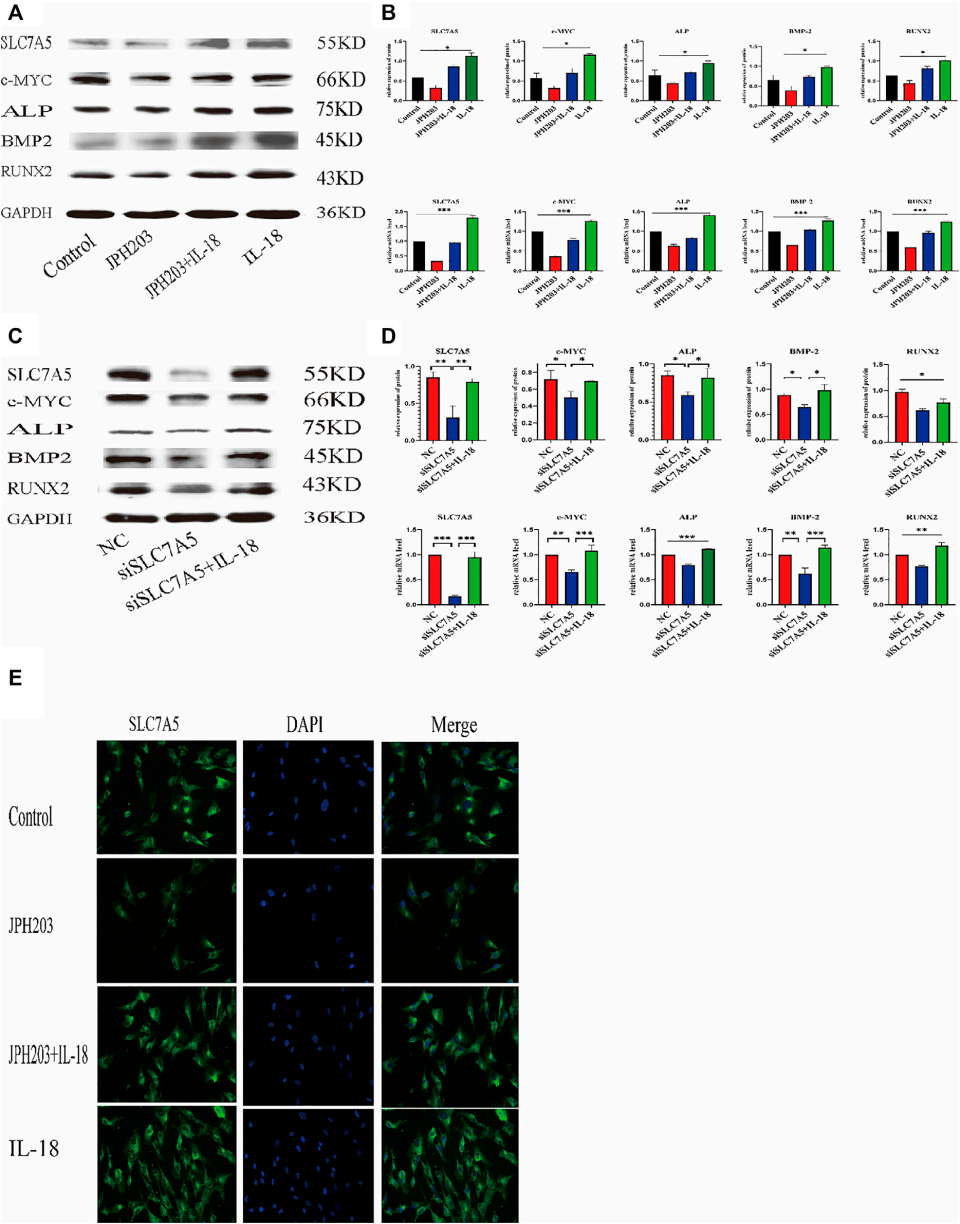
Inhibition of SLC7A5 reduces the bone-forming ability of hBMSCs induced by IL-18. The cells were treated with JPH203 (5 μM) for 48 h, and then with 100 ng/ml IL-18 for 12 h western blotting and q-PCR experiments were conducted to evaluate the expression of osteoblast-specific markers, as well as SLC7A5 and c-MYC mRNA; GAPDH was used as an internal control **(A, B)**. Cells were transfected for 48 h and then treated with 100 ng/ml IL-18 for 12 h, mRNA levels of SLC7A5, c-MYC and osteoblast-specific factors were quantied by qRT-PCR ,Cells were transfected for 72 h and then treated with 100 ng/ml IL-18 for 12 h and then western blotting experiments were conducted to evaluate the expression of osteoblast-specific factors, as well as SLC7A5 and c-MYC; GAPDH was used as an internal control **(C, D)**.Cells were treated with JPH203 (5 μM) and IL-18 (100 ng/ml) for 48 h, and the protein levels of SLC7A5 were assessed by immunofluorescence **(E)**. Data expressed as the mean ± standard deviation (SD). Experiments were repeated three times. Scale bars: 200 μm for F, G. *p < 0.05, **p < 0.01, ***p < 0.001 (multiple comparisons use one-way ANOVA with a subsequent Bonferroni post-hoc test).

To investigate whether SLC7A5 is involved in the IL-18-induced osteogenic differentiation of hBMSCs, we transfected cells with siSLC7A5 to specifically downregulate the expression of SLC7A5. In another set of experiments, we used JPH203, a SLC7A5 specific inhibitor, to evaluate the role of SLC7A5 in the osteogenic differentiation of hBMSCs. Our results demonstrated that, compared to the control group, the expression levels of SLC7A5 and c-MYC in the siSLC7A5 and JPH203 treatment groups were significantly reduced, and the expression levels of osteogenic markers ALP, BMP2, and RUNX2 were also decreased (Figures 5A–D). Furthermore, our results showed that IL-18-induced SLC7A5 expression could be reversed either by JPH203 treatment or siSLC7A5. As to the immunofluorescent staining results, compared with the IL-18 + JPH203 group, the IL-18 group showed the highest expression levels, and the JPH203 group showed the lowest expression levels (Figure 5E).

### Osteogenic Differentiation Ability of hBMSCs That Inhibit the Decrease of c-MYC Expression can Be Enhanced by IL-18

**FIGURE 6:**
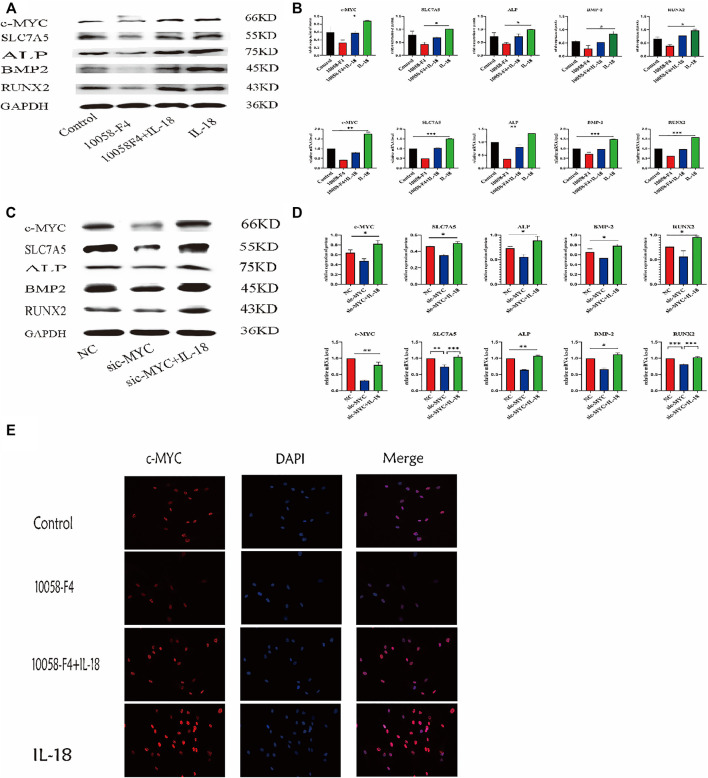
Inhibition of c-MYC can reduce the bone-forming ability of HBMSC induced by IL-18. The cells were treated with 10058-F4 (5 μM) for 72 h, and then with 100 ng/ml IL-18 for 12 h western blotting and q-PCR experiments were conducted to detect osteogenic factors and the protein and mRNA expression of c-MYC and SLC7A5, ALP, BMP2, RUNX2; GAPDH was used as an internal control **(A, B)**. Cells were transfected for 48 h and then treated with 100 ng/ml IL-18 for 12 h, mRNA levels of SLC7A5, c-MYC and osteoblast-specific factors were quantied by qRT-PCR, Cells were transfected for 72 h and then treated with 100 ng/ml IL-18 for 12 h and then western blotting experiments were conducted to evaluate the expression of osteoblast-specific factors, as well as c-MYC and SLC7A5; GAPDH was used as an internal control **(C, D)**. After treatment with 10058-F4 (5 μM) for 72 h and 100 ng/ml IL-18 for 12 h, immunofluorescence staining was used to observe c-MYC protein levels **(E)**. Data expressed as the mean ± standard deviation (SD). Experiments were repeated three times.*p < 0.05, **p < 0.01, ***p < 0.001 (multiple comparisons use one-way ANOVA with a subsequent Bonferroni post-hoc test).

To further investigate whether the inhibition of c-MYC can reverse the IL-18-induced osteogenic differentiation of hBMSCs, cells were treated with c-MYC antagonists 10058-F4 and sic-MYC to inhibit c-MYC expression. Compared to the control group, 10058-F4 and sic-MYC significantly reduced the expression of c-MYC at the mRNA and protein levels, with expression of c-MYC reduced, it’s also reduced the expression of SLC7A5,ALP,BMP2 and RUNX2; however, IL-18 treatment partially restored the bone formation ability in 10058-F4 and sic-MYC groups (Figures 6A–D). Immunofluorescent staining also showed that IL-18 promoted the nuclear expression of c-MYC, while 10058-F4 significantly reduced the nuclear expression of c-MYC that was induced by IL-18 (Figure 6E).

## Discussion

Currently, an increasing number of studies are involved in the investigation of the role of immune response mechanisms during fracture healing. It has been reported that, within 24 h of an injury, the expression of cytokines is increased in the fracture hematoma; however, the role of cytokines in these hematomas has not been fully understood. Here we showed that IL-18 induced the mineralization and osteogenic differentiation of hBMSCs at a biologically relevant concentration, which is crucial for the investigation of its molecular mechanism *in vitro*. Our results also indicated that 100 ng/ml of IL-18 induced the strongest osteogenic effect. We also tested 120 ng/ml concentration of IL-18 in the cells; however, this concentration was found to be toxic to the cells, and cells died and floated within a few minutes. Therefore, 100 ng/ml was considered to be the optimal concentration. Our results demonstrated that SLC7A5 and c-MYC played an important role in the IL-18-induced expression of osteogenic markers in hBMSCs; IL-18 upregulated the expression of SLC7A5 and c-MYC at the early stage of hBMSC osteogenic differentiation, and SLC7A5 and c-MYC inhibition blocked the osteogenic differentiation that was induced by IL-18. To the best of our knowledge, this is the first report demonstrating that SLC7A5 is involved in the activation of c-MYC in hBMSCs (Figure 3, Figure 5).

Previous studies have shown that the levels of IL-18 in the blood are significantly increased after a fracture, while the levels of IL-18 return to normal levels after the fracture is repaired (Ko et al., 2018; Li and Wang, 2018). We previously demonstrated that the levels of proinflammatory factors in hematoma and peripheral blood are significantly increased after a fracture. Other studies have shown that IL-18 induces the expression of osteoprotegerin (OPG) in mouse osteoblasts to inhibit the formation of osteoclasts (Makiishi-Shimobayashi et al., 2001). The bone density of elderly patients with osteoporosis increases significantly after anti-osteoporosis treatment. This may be related to the ability of IL-18 to inhibit osteoclast activity, induce the proliferation and differentiation of bone marrow-derived lymphoid progenitor cells, and promote NK cell proliferation and cytotoxicity (Maugeri et al., 2005; Gandhapudi et al., 2015; Choi et al., 2019). These findings suggest that IL-18 has some beneficial impacts; however, it has a negative effect on rheumatoid arthritis (RA) and osteoarthritis (OA). For example, IL-18 induces inflammatory responses in synovial cells and chondrocytes (Fu et al., 2012); however, the upregulation of splenic suppressors of cytokine signaling (SOCS) can reduce the release of pro-inflammatory cytokine IL-18 and relieve the symptoms of RA. It is possible that, in these two chronic diseases, the continued release of IL-18 leads to abnormal bone formation (Nozaki et al., 2019), while during bone fracture, an acute condition with an early phase lasting approximately 3 days, pro-inflammatory factors and immune cells in the fractured hematoma tissue promote hBMSC migration and fracture healing (Pountos et al., 2019). However, the effect of IL-18 on other bone-related factors in hBMSCs is still unclear. Here, in this study, we observed that IL-18 can induce the expression of osteogenic factors, such as ALP, BMP2, and RUNX2, in a dose-dependent manner. At the same time, IL-18 (100 ng/ml) can promote mineralization, confirming the osteogenic differentiation of hBMSCs (Figure 1). Runx2 is a transcription factor that regulates the expression of early osteoblast-specific genes, while BMP-2 is a growth factor known to induce osteoblast differentiation and the expression of osteogenic genes, and therefore plays an important role in bone repair (Salazar et al., 2016). ALP is another osteoblast-specific factor involved in the regulation of bone morphogenesis by generating the phosphoric acid necessary for the deposition of hydroxyapatite during mineralization. The higher the ALP activity, the stronger the osteogenic differentiation ability (Wang et al., 2018). Therefore, our results confirm that the regulation of cell surface molecules by pro-inflammatory cytokines after fracture plays an important role in the activation of BMSCs. Accurately increasing the levels of IL-18 at the fracture site after fracture, in combination with splenectomy, may provide a new treatment strategy for fracture healing in the early stage of fracture.

Amino acids can affect the proliferation, differentiation, and mineralization of BMSCs through intracellular oxidative stress and the tricarboxylic acid cycle (TCA) cycle (Dirckx et al., 2019; Zhou et al., 2019). Furthermore, a low essential amino acid diet in mice can lead to bone loss and osteoporosis. SLC7A5 is an essential amino acid transporter that plays a key role in cell metabolism and growth. The importance of SLC7A5 was demonstrated using an animal global knockout model, with the embryos not surviving past the second trimester. Furthermore, BMSCs grown in a medium with low amino acid levels have significantly reduced proliferation and differentiation ability. At the same time, the abnormal expression of SLC7A5 can cause diseases, such as tumors, Parkinson’s, neurodevelopmental abnormalities, and autism, confirming that SLC7A5 plays a crucial role in physiological processes (Tărlungeanu et al., 2016; Ding et al., 2018; Scalise et al., 2018). The expression of SLC7A5 in osteoclasts of a mouse osteoporosis model was reportedly significantly reduced. By regulating nuclear factor of activated T cells, cytoplasmic 1 (NFATc1) in osteoclasts, it plays a key role in bone resorption and bone homeostasis. It was shown that SLC7A5 knockout mice developed osteoporosis. The femur bone density was significantly reduced, while the levels of osteoclast markers in the blood were significantly increased (Ozaki et al., 2019), confirming that SLC7A5 plays an important role in bone homeostasis. However, the specific role of SLC7A5 in BMSCs is not clear. Several studies have shown that stem cells express a variety of amino acid transporters, while high expression levels of SLC7A5 and energy metabolism are essential for the osteogenic differentiation of hBMSCs (Moravcikova et al., 2018). Our results, using hBMSCs, showed that in response to IL-18 treatment, SLC7A5 expression increased in a concentration-dependent manner, while the expression levels of SLC7A5 were significantly decreased by SLC7A5 inhibitors and siRNA.

SLC7A5 is necessary for the growth of T cells. T cells lacking SLC7A5 do not undergo metabolic reprogramming, expansion, and differentiation in response to antigen stimulation. Furthermore, SLC7A5 knockout mouse embryos have serious nerve and limb growth defects (Sinclair et al., 2013; Poncet et al., 2020). Another study demonstrated that in tumor cells SLC7A5 can regulate the expression of c-MYC, forming a mechanism that connects essential amino acid transport and tumorigenesis. The positive feedback loop mechanism is probably due to the fact that tumor cells have higher energy and nutrient demands to maintain cell survival and proliferation (Yue et al., 2017). Here, in our study, we used SLC7A5 inhibitor JPH203 and siSLC7A5 to investigate whether IL-18 promoted the osteogenic differentiation of hBMSCs *via* SLC7A5. We found that JPH203 and siSLC7A5 significantly inhibited the expression of IL-18-induced osteogenic markers, such as Runx2, ALP, and BMP2, and significantly reduced the expression of c-MYC. These results suggested that IL-18 induced the osteogenic differentiation of hBMSCs *via* SLC7A5 (Figure 5). These findings are consistent with previous studies demonstrating osteoporosis in SLC7A5 knockout mice. JPH203 is a selective inhibitor of SLC7A5 which can effectively block the amino acid transport mediated by SLC7A5 (Enomoto et al., 2019; Ozaki et al., 2019).

Recent studies have shown that c-MYC is a downstream target gene of multiple signaling pathways and plays key roles in numerous physiological processes, such as embryonic development, self-renewal of stem cells, and tissue regeneration, as well as cellular differentiation (Liu et al., 2017). Under normal physiological conditions, the expression of c-MYC is strictly regulated and increases in response to extracellular growth factors; in response to these growth factors, c-MYC is quickly activated (Meyer and Penn, 2008). The expression of c-MYC in osteoblast culture medium is significantly increased. c-MYC is widely regarded as a marker of pluripotent stem cells such as ESCs and iPSCs and is highly expressed in stem cells, and regulates the pluripotency of mouse ESCs, neural stem cells (NSC), and hematopoietic stem cells (HSC). The key role of sex and self-renewal ability is known (Nair et al., 2014; Foroutan, 2016), but research on the specific mechanism of action is limited. Studies have found that β-catenin signaling can upregulate the expression of OSX in human pre-osteoblasts and bone marrow stromal cells through c-MYC to promote osteogenesis. These findings suggest that c-MYC plays an important role in osteogenesis (Liu et al., 2015). The c-MYC pathway also plays a key role in the maintenance of T cell function and metabolic reprogramming and is independent of the mTOR pathway. The physiological condition, in terms of the transcription levels of c-MYC, reflects tissue and cell growth status; tissues with high proliferation rates have high c-MYC transcription levels (Lu et al., 2020). Several studies have demonstrated that many types of tumors are characterized by high c-MYC expression levels; however, in the absence of other mutations, c-MYC overexpression alone is not sufficient for tumorigenic transformation. These findings suggest that c-MYC plays a pivotal role in physiological and pathological conditions (Wolfer and Ramaswamy, 2011; Cascón and Robledo, 2012; Sinclair et al., 2013).

Whole genome analysis of rat fibroblasts showed that SLC7A5 is the direct action site of c-MYC. The increase of c-MYC activity upregulates the expression of several glutamine transporters, and in c-MYC knockout mice resulted in embryonic death and significantly reduced SLC7A5 levels (Zhao et al., 2019). Previous studies have shown that c-MYC enhances the expression of SLC7A5 by binding to specific promoters (Gao et al., 2009; Hayashi et al., 2012). Other studies have also found that c-MYC in cells can be activated by directly binding to specific E-box sequences to initiate SLC7A5 transcription, while SLC7A5-mediated uptake of essential amino acids stimulates c-MYC protein synthesis and downstream target gene transcription, leading to reprogramming of the entire metabolic process, including glycolysis, glutamine breakdown, and lipogenesis (Yue et al., 2017). In tumor cells, the expression of c-MYC has been shown to significantly decrease after the JPH203-mediated inhibition of SLC7A5, thereby affecting the metabolic function controlled by c-MYC (Rosilio et al., 2015). It has also been shown that high expression levels of c-MYC appear to be essential for the maintenance of MSC proliferation and differentiation potential. However, low c-MYC expression or loss of function leads to the inhibition of MSC proliferation and differentiation. It was recently demonstrated that c-MYC overexpression increases type X collagen one (COL10A1) expression, suggesting that c-MYC plays an important role in cartilage formation (Wang et al., 2017; Melnik et al., 2019). These findings indicate that c-MYC is an important regulatory factor in stem cells and are consistent with our results. Here we showed that the use of siRNA and c-MYC inhibitor 10058-F4 resulted in the downregulation of SLC7A5 expression, as well as of osteogenic markers, indicating that c-MYC inhibition can decrease the osteogenic differentiation ability of hBMSCs *in vitro*. Moreover, IL-18 reversed the downregulation of c-MYC and osteogenic markers induced by siRNA and the c-MYC specific inhibitor 10058-F4, indicating that IL-18 plays a role *via* c-MYC regulation (Figure 6). Our study determined the close relationship between the SLC7A5 and c-MYC signaling pathway and the expression of Runx2, BMP2, and ALP, as well as the close relationship between SLC7A5 and c-MYC. The results indicate that IL-18 promotes the osteogenic differentiation of hBMSCs through the SLC7A5/c-MYC regulatory axis. Therefore, activating the SLC7A5/c-MYC axis can promote fracture healing after splenectomy.

Our results show that IL-18 promotes bone formation *in vitro*. It activates SLC7A5 to enhance the osteogenic differentiation of hBMSCs mainly through the c-MYC pathway. Blocking SLC7A5/c-MYC reduces the osteogenic differentiation of hBMSCs, indicating that the SLC7A5/c-MYC axis plays an important role in the osteogenic differentiation of hBMSCs, suggesting that the spleen plays an important role in the fracture healing process. Therefore, when fractures with splenic injury require surgical treatment, the spleen should be preserved as much as possible during the operation to ensure stability of the patient’s immune function after surgery. These findings will provide new treatment strategies for delayed fracture healing after splenectomy.

## Data Availability

The original contributions presented in the study are included in the article/Supplementary Materials, further inquiries can be directed to the corresponding author.
